# Association of Periodontitis with Mild Cognitive Impairment in Older Adults

**DOI:** 10.14283/jarlife.2024.16

**Published:** 2024-12-04

**Authors:** M. Igase, K. Igase, S. Hino, D. Uchida, Y. Okada, M. Ochi, Y. Tabara, Y. Ohyagi

**Affiliations:** 1. Department of Anti-Aging Medicine, Ehime University Graduate School of Medicine, Toon City, 791-0295, Japan; 2. Department of Advanced Neurosurgery Ehime University Graduate School of Medicine, Toon City, 791-0295, Japan; 3. Department of Oral and Maxillofacial Surgery, Ehime University Graduate School of Medicine, Toon City, 791-0295, Japan; 4. Department of Geriatric Medicine and Neurology, Ehime University Graduate School of Medicine, Toon City, 791-0295, Japan; 5. Graduate School of Public Health, Shizuoka Graduate University of Public Health, Kita-Ando 4-27-2, Aoi-ku, Shizuoka, 420-0881, Japan

**Keywords:** Periodontitis, mild cognitive impairment, MCI screen, probing pocket depth, multivariate logistic regression model

## Abstract

**Background:**

Early detection of cognitive decline, including mild cognitive impairment, is expected to provide a better prognosis. Several studies have suggested an association between periodontitis and mild cognitive impairment.

**Objectives/Design:**

To test the hypothesis that there is an association between severe periodontitis and mild cognitive impairment in community residents who participated in a dental health check-up program.

**Participants/Setting:**

Community residents who participated in our dental health checkup program were enrolled (age=67.5±9.9, 62.9% female).

**Measurements:**

Mild cognitive impairment was tested using the MCI screening test. Periodontitis was diagnosed based on a widely used clinical periodontal parameter, the probing pocket depth. Statistical analysis was based on logistic regression models adjusted for potential confounders.

**Results:**

Among 321 subjects, mild cognitive impairment was detected in 41. Severe periodontitis (probing pocket depth > 6mm) was detected in 123 cases, with a higher prevalence of mild cognitive impairment in the severe periodontitis group (65.9%) than in the unimpaired group (34.3%). The inclusion of four variables (age, education, functional teeth, and presence of severe periodontitis) in a multivariate logistic regression model revealed a statistically significant difference in the association between severe periodontitis and mild cognitive impairment (odds ratio = 4.024, p < 0.001).

**Conclusions:**

A strong association was seen between severe periodontitis and mild cognitive impairment. Severe periodontitis appears to be a risk factor for mild cognitive impairment, and patients with severe periodontitis should be assessed for mild cognitive impairment.

## Introduction

**D**ementia is a neurodegenerative disease syndrome of the central nervous system in the elderly (1). Alzheimer disease (AD) is the leading cause of age-related dementia, reported to affect approximately 57.4 million people worldwide in 2021 (2). AD is a disease with multiple etiologies and complex pathology. Effective therapy for AD is still in its early stages and usually demonstrates limited curative effect (3).

Early detection of AD in an earlier phase of cognitive decline is expected to provide a better prognosis (4). Recent attention has focused on patients with mild cognitive impairment (MCI), who are at high risk for developing dementias, including AD (5). MCI is representing the stage between the age dependent decline in memory and thinking and the more serious decline of dementia. In general, approximately 10% to 15% of MCI cases progress to AD each year (6).

Periodontitis is a chronic infectious disease of the oral cavity that manifests as the progressive destruction of the supporting tissues of the teeth (7). Also, periodontitis is a common source of systemic inflammation and especially neuroinflammation might be a result of this to accelerate progressive deterioration of neuronal functions during aging or exacerbate pre-existing neurodegenerative diseases, such as Alzheimer’s disease (AD).

Recent study showed that IL-1β and TNF-α were upregulated upon periodontitis and the systemic upregulation of these two cytokines may promoted a pro-inflammatory environment in the brain contributing to the development of AD (8). Given these results, it is considered that systemic inflammation serves as a connecting link between periodontitis and AD.

Probing pocket depth (PPD) is a dental procedure that measures the depth of the pocket between the tooth and the gum line and in general, the average healthy PPD is around 3 mm. Although with severe AD have been found to have significantly higher PPD values than those with mild AD (9).

In this study, we sought to establish an attributable risk of periodontitis as possible trigger for MCI using data from participants in our health checkup program and mitigate the possible routes of AD onset. In addition, several studies have suggested an association between periodontitis and MCI (10, 11). Although periodontitis might affect the development of cognitive impairment through different mechanisms, the question of whether periodontitis is a risk factor for mild cognitive impairment (MCI) in the general population remains uncertain.

## Methods

### Inclusion and exclusion criteria

The subjects were participants in a complete medical checkup program at the Ehime University Hospital Anti-aging Center (Ehime, Japan), which is specifically designed to evaluate for atherosclerosis, including cerebrovascular disease (12) and oral health status. This program is provided for community residents without specific requirements for participation and aims to evaluate factors relating to dementia. As for oral health status, applicants can select this. Participants who gave informed consent to the use of clinical information obtained at the health checkup program for a longitudinal study were enrolled in the Shimanami Health Promoting Program (the J-SHIPP study) conducted by the Ehime University Graduate School of Medicine. On behalf of all authors, the corresponding author states that there is no conflict of interest.

Of 324 participants who participated in the dental oral health checkup program between February 2016 and March 2023, we excluded one participant with no functional teeth and two participants those with a score of < 24 on the Mini-Mental State Examination (MMSE).

### Assessment for MCI

To discriminate mild from severe cognitive impairment, we used the Mini-Mental State Examination (MMSE). This test is the most often-used short screening tool to obtain an overall measure of cognitive impairment in clinical, research and community settings and is the most commonly used cognitive screening test for MCI and dementia (13, 14). Maximum score is 30 points, and the 24-point cut-off score is considered to demonstrate good specificity and acceptable sensitivity (15).

### MCI screening

MCI was assessed for using the Japanese version of the MCI screening test, a 10-min, computationally scored, staff-administered test (16). Cross-validation was performed using the Clinical Dementia Rating score as reference. Overall accuracy of this score in identifying subjects with MCI is 97% (17).

### Education

All participants completed a questionnaire regarding the highest level of education achieved.

### Blood sample collection

Blood samples were collected between 09.00 and 10.00 hours from the cubital vein after an overnight fast. HbA1c level was determined in fresh samples.

### Periodontitis diagnosis

The diagnosis of periodontitis was based on a widely used clinical periodontal parameter, the PPD (18).

### Statistical methodology

We performed a cross-sectional study looking for possible associations between periodontitis and MCI.

All continuous variables are expressed as mean ± SD, unless otherwise indicated. The normal distribution (Kolmogorov-Smirnov test) and the homoscedasticity (Levene test) of the data were verified. Comparisons between the MCI and normal groups were assessed using the unpaired t-test for parametric variables and the Mann-Whitney U-test for nonparametric variables. The chi-squared test was used to assess frequency differences between the number of MCI cases and number of male subjects. Covariate adjusted analysis was performed by multiple logistic regression analyses with possible independent parameters including age, Hba1c, education, functional teeth, PPD, and the presence of severe periodontitis. In all comparisons, a p value < 0.05 was considered statistically significant. Correlations between variables were evaluated using Pearson’s correlation coefficient. Analyses were performed using commercial software (SPSS software package for Windows version 17, SPSS, Chicago, IL, USA).

## Results

Clinical characteristics of the MCI group and normal cognition group are summarized in Table 1. Among 321 cases, MCI was detected in 41 cases (12.8%), of which 18 (43.9%) were male. The MCI group was significantly older (74.8 ± 8.5 y) than the normal group (66.3 ± 9.5 year; p <0.001) and had significantly fewer years of education (12.0 ± 2.4 y) than the normal group (13.2 ± 2.1 y; p = 0.002). Other known risk factors for MCI, including sex, BMI, and HbA1c, did not significantly differ between the MCI and normal groups.

**Table 1.** Clinical characteristics of the MCI group and normal cognition group

	All (n = 321)	MCI (n = 41)	Normal (n = 280)	p
Age (years)	67.5 ± 9.9	74.8 ± 8.5	66.3 ± 9.5	< 0.001
Sex (men/women)	119/202	18/23	101/179	0.212
BMI (kg/m2)	22.6 ± 3.1	23.0 ± 3.3	22.5 ± 3.1	0.322
HbA1c (%) *	5.72 ± 0.69	5.77 ± 1.30	5.71 ± 0.6	0.646
Education (years)	13.0 ± 2.1	12.0 ± 2.4	13.2 ± 2.1	0.002
MPI (points)	61.8 ± 11.5	42.7 ± 7.8	64.9 ± 8.8	< 0.001
MMSE (points)	29.1 ± 2.0	25.6 ± 2.8	29.7 ± 1.0	< 0.001
Functional teeth (n)	26.0 ± 3.1	24.2 ± 3.4	26.3 ± 3.0	0.004
PPD > 4 mm (n)	9.7 ± 7.5	12.1 ± 7.3	9.5 ± 7.5	0.039
PPD > 4 mm(%)	41.9 ± 30.8	56.6 ± 30.3	40.1 ± 30.2	0.001
Max PPD (mm)	5.3 ± 1.6	6.1 ± 1.4	5.2 ± 1.5	0.001
Severe periodontitis, n (%)	123 (38.3)	27 (65.9)	96 (34.3)	< 0.001

MCI, Mild Cognitive Impairment; BMI, body mass index; HbA1c, glycosylated hemoglobin; MPI, Memory performance index; MMSE, Mini-Mental State Examination; PPD; probing pocket depth. *HbA1c: equivalent to the internationally used HbA1c defined by the National Glycohemoglobin Standardization Program.

There was a significant difference between the MCI and normal group in the number of functional teeth (26.0 ± 3.1 vs. 24.2 ± 3.4, p = 0.004). The incidence of MCI was not significantly higher in patients with mild to moderate periodontitis (odds ratio [OR] = 1.042; 95% confidence interval [CI]: 0.999–1.087 p = 0.054) (Table 2). In contrast, the percentage of teeth affected by periodontitis (PPD > 4 mm) was significantly higher in the MCI than in the normal group (p=0.001). In addition, severe periodontitis (PPD > 6 mm) was detected in 123 patients (38.3%) and the percentage of MCI was significantly higher in the severe periodontitis group than in the normal group (p < 0.001).

**Table 2.** Multiple logistic regression analysis to assess the presence of MCI

	Crude OR (95%CI)	p	Adjusted OR (95%CI)	p
Age	1.122 (1.073–1.174)	< 0.001	1.126(1.064–1.191)	< 0.001
Hba1c	1.079 (0.690–1.687)	0.738		
Education, y	0.759 (0.635–0.907)	0.002	0.809 (0.672–0.974)	0.025
Functional teeth, n	0.943 (0.904-0.985)	0.008	1.015 (0.950–1.085)	0.650
PPD > 4 mm, n	1.042 (0.999–1.087)	0.054		
PPD > 4 mm, %	1.017 (1.007–1.029)	0.002		
Severe periodontitis	3.696 (1.852–7.377)	< 0.001	4.024 (1.768–9.161)	< 0.001

OR = odds ratio; CI = confidence interval; PPD = probing pocket depth

Four variables (age, education, number of functional teeth, and severe periodontitis) were entered into a multivariate logistic regression model. A statistically significant difference was seen in the association between severe periodontitis and MCI (OR = 4.024, 95% CI: 1.768–9.161, p < 0.001) (Table 2).

## Discussion

In this study of subjects of a medical/dental checkup program, we identified severe periodontitis in 123 of 321 examinees. The prevalence of MCI was significantly higher in the severe periodontitis group than in the normal groups. Multivariate logistic regression analysis revealed a statistically significant difference in the association between severe periodontitis and MCI. These findings suggest that severe periodontitis may be a risk factor for MCI, and that affected patients should be assessed for mild cognitive impairment.

Because poor oral health is highly suspected to be a risk factor for dementia (19), the correlation between clinical oral condition, including the number of functional teeth and severity of periodontitis, and cognitive decline has now become a research concern. Our investigation of the effects of the number of functional teeth and severity of periodontitis on mild cognitive impairment (MCI) provides scientific evidence for further research.

### Association between number of functional teeth, periodontitis and cognitive decline

In their cross-sectional study of the number of functional teeth in AD patients, Tsuneishi et al. reported that older people visiting dental offices with fewer teeth were more likely to have AD (20).

With regard to MCI, our study found a significant difference between MCI patients and normal patients in both the number of functional teeth and presence or absence of periodontitis (functional teeth: 24.2 ± 3.4 in the MCI group vs. 26.3 ± 3.0 in the normal group. p=0.004; presence of periodontitis: 12.1 ± 7.3 in the MCI group vs. 9.5 ± 7.5 in the normal group. p= 0.039). Luo H et al showed that the absence of ≥ 8 or more functional teeth was a significant risk factor for MCI, and suggested that better access to dental care, health education on risk factors of MCI, and promotion of good oral health may mitigate the burden of cognitive impairment (21). Their results are concordant with our present study.

### Association between periodontitis and cognitive decline

Tiisanoja et al. demonstrated that when periodontitis was defined as more than 1 tooth with a PPD > 4 mm, the relative risks of periodontitis and dementia did not significantly differ (22).

A recent meta-analysis also showed no statistical significance in the effect of mild periodontitis on dementia (OR = 1.59; 95%CI, 0.92–2.76). However, subgroup analysis revealed that moderate or severe periodontitis was significantly associated with dementia (OR = 2.13; 95%CI, 1.25–3.64) (23). Consistent with these findings, we also identified a slight but significant difference between the MCI group and normal group regarding the number of periodontitis-affected teeth, but did not find a significantly higher incidence of MCI in patients with mild to moderate periodontitis. However, the percentage of MCI was significantly higher in the severe periodontitis group than in the normal groups. Finally, we demonstrated using multivariate logistic regression analysis that severe periodontitis was an independent risk factor for the presence of MCI, with a greater than fourfold higher OR of MCI in those with severe periodontitis than in those without this condition.

The search for effective predictors of dementia in people with MCI has attracted strong interest. Our present and previous study results indicate that patients with severe periodontitis are at higher risk of developing MCI, followed by dementia. Periodontitis can be alleviated and improved through self-management and medical examination. Considering the treatability of periodontitis, prospective analysis of the association between periodontitis treatment and MCI may be useful.

Several limitations of this study are noteworthy. First, routine screening programs in most countries do not include a medical/dental checkup program. In Japan, in contrast, medical/dental checkup programs are widely available to the general public. Our institution’s medical/dental checkup program provides extensive discretionary screening. However, it is needed to recognize that there was high selection bias in our study. Because the body size of Asians, including Japanese, is smaller than that of Westerners (24), our findings may not be entirely generalizable to other populations. Second, although we used the MCI screen in this study, the Montreal Cognitive Assessment (MoCA) test is reported to better meet the criteria for screening for detection of MCI among patients aged over 60 years (25). If we had also used the MoCA test, the accuracy of our results may have improved. Third, with regard to MCI assessment, our analyses were retrospective, and a degree of selection bias may have been introduced. Study participants were recruited from antiaging checkup examinees, and accordingly the participants may not necessarily have represented the general population. In particular, our program participants were fitness-conscious, and heavy smokers and alcohol drinkers may have been underrepresented. Fourth, the cross-sectional nature of the study prevents any assignation of causality in the association between periodontitis and mild cognitive impairment. Moreover, we were unable to assess the association of periodontitis with dementia as no program participant had dementia. Allowing for these limitations, we found a strong association between severe periodontitis and MCI.

## References

[1] Ferri CP, Prince M, Brayne C, et al. Global prevalence of dementia: a Delphi consensus study. Lancet 2005; 366: 2112-2117. doi: 10.1016/S0140-6736(05)67889-0. 16360788 PMC2850264

[2] Gauthier S, Rosa-Neto P, Morais JA, Webster C. World Alzheimer Report 2021: Journey through the Diagnosis of Dementia. Alzheimer’s Disease International; London, UK: 2021.

[3] Duan LH, Li LI, Wang CB, Liu QQ, Zhang X, Wu ZZ. Brain targeting efficacy of novel drug delivery system in the treatment of Alzheimer’s disease. Eur Rev Med Pharmacol Sci. 2024; 28: 3892-3904. doi: 10.26355/eurrev_202407_36521. 39012229

[4] Raja HA, Nashwan AJ. Predictors of prognosis in Alzheimer’s disease: The role of cognitive dysfunction, immune abnormalities, and advanced neuroimaging. World J Clin Cases. 2024 Sep 26;12(27):6004-6006. doi: 10.12998/wjcc.v12.i27.6004. 39328849 PMC11326107

[5] van Dyck C. H., Swanson CJ., Aisenet P., et al. Lecanemab in Early Alzheimer’s Disease. The New Engl. J. Medicine 2023; 388, 9–21, DOI: 10.1056/NEJMoa2212948(2023). 36449413

[6] Petersen RC, Smith GE, Waring SC, Ivnik RJ, Kokmen E, Tangelos EG. Aging, memory, and mild cognitiveimpairment. Int Psychogeriatr. 1997; 9 Suppl 1: 65-9. doi: 10.1017/s1041610297004717. 9447429

[7] Davatzikos C, Bhatt P, Shaw LM, Batmanghelich KN, Trojanowski JQ. Prediction of MCI to AD conversion, via MRI, CSF biomarkers, and pattern classification. Neurobiol. Aging. 2011; 32: 2322–e19. doi: 10.1016/j.neurobiolaging.2010.05.023. PMC2951483 20594615

[8] Wang RP, Huang J, Chan KWY, et al. IL-1beta and TNF-alpha play an important role in modulating the risk of periodontitis and Alzheimer’s disease. J Neuroinflammation. 2023; 20: 71. doi: 10.1186/s12974-023-02747-4. 36915108 PMC10012546

[9] Tonetti MS, Greenwell H, Kornman KS. Staging and grading of periodontitis: framework and proposal of a new classification and case definition. J Periodontol. 201889 Suppl 1S15972. 10.1002/JPER.18-0006 29926952

[10] Borsa L, Dubois M, Sacco G, Lupi L. Analysis [sic] the link between periodontal diseases and Alzheimer’s disease: a systematic review. Int J Environ Res Public Health. 2021; 18: 9312. doi: 10.3390/ijerph18179312. 34501899 PMC8430572

[11] Karaduran K, Aydogdu A, Gelisin O, Gunpinar S. Investigating the potential clinical impact of periodontitis on the progression of Alzheimer’s disease: a prospective cohort study. Clin Oral Investig. 2023; 28: 67. doi: 10.1007/s00784-023-05445-w. 38159159

[12] Holmer J, Eriksdotter M, Schultzberg M, Pussinen PJ, Buhlin K. Association between periodontitis and risk of Alzheimer’s disease, mild cognitive impairment and subjective cognitive decline: a case-control study. J Clin Periodontol. 2018; 45: 1287–98. doi: 10.1111/jcpe.13016. 30289998

[13] Lin ZK, Ma SJ, Qian JL, et al. Association between periodontitis and mild cognitive impairment: a clinical pilot study. Zhonghua Kou Qiang Yi Xue Za Zhi. 2022; 57: 576–84. doi: 10.3760/cma.j.cn112144-20220414-00181. 35692001

[14] Okada Y, Kohara K, Ochi M, et al. Mechanical stresses, arterial stiffness, and brain small vessel diseases: Shimanami Health Promoting Program Study. Stroke. 2014; 45: 3287–3292. doi: 10.1161/STROKEAHA.114.006539. 25228261

[15] Folstein MF, Folstein SE, McHugh PR. “Mini-mental state”. A practical method for grading the cognitive state of patient for the clinician. J Psychiatr Res. 1975; 12: 189–198. doi: 10.1016/0022-3956(75)90026-6. 1202204

[16] Arevalo-Rodriguez I, Smailagic N, et al. Mini-Mental State Examination (MMSE) for the detection of Alzheimer’s disease and other dementias in people with mild cognitive impairment (MCI). Cochrane Database Syst Rev. 2015; 2015. doi: 10.1002/14651858.CD010783.pub2. PMC6464748 25740785

[17] Tombaugh TN, McIntyre NJ. The Mini-Mental State Examination: a comprehensive review. Journal of the American Geriatrics Society 1992; 40: 922-935. doi: 10.1111/j.1532-5415.1992.tb01992.x. 1512391

[18] Salis F, Costaggiu D, Mandas A. Mini-Mental State Examination: Optimal Cut-Off Levels for Mild and Severe Cognitive Impairment. Geriatrics (Basel) 2023; 8: 12. DOI: 10.3390/geriatrics8010012. 36648917 PMC9844353

[19] Cho A, Sugimura M, Nakano S, Yamada T. The Japanese MCI screen for early detection of Alzheimer’s disease and related disorders. Am J Alzheimers Dis Other Demen 2008; 23: 162–166. doi: 10.1177/1533317507312624. 18223126 PMC10846169

[20] Trenkle DL, Shankle WR, Azen SP. Detecting cognitive impairment in primary care: performance assessment of three screening instruments. J Alzheimers Dis 2007; 11: 323–335. doi: 10.3233/jad-2007-11309. 17851183

[21] Harase T, Nishida W, Hamakawa T, et al. Clinical implication of blood glucose monitoring in general dental offices: the Ehime Dental Diabetes Study. BMJ Open Diabetes Res Care. 2015; 3: e000151. doi: 10.1136/bmjdrc-2015-000151. PMC4653862 26629348

[22] Daly B, Thompsell A, Sharpling J, et al. Evidence summary: The relationship between oral health and dementia. Br Dent J. 2018; 223: 846–853. doi: 10.1038/sj.bdj.2017.992. 29192686

[23] Tsuneishi M, Yamamoto T, Yamaguchi T, Kodama T, Sato T. Association between number of teeth and Alzheimer’s disease using the National Database of Health Insurance Claims and Specific Health Checkups of Japan. PLoS One. 2021; 16: e0251056. doi: 10.1371/journal.pone.0251056. 33930067 PMC8087029

[24] Luo H, Wu B, González HM, et al. Tooth Loss, Periodontal Disease, and Mild Cognitive Impairment Among Hispanic/Latino Immigrants: The Moderating Effects of Age at Immigration. J Gerontol A Biol Sci Med Sci. 2023; 78: 949–957. doi: 10.1093/gerona/glac178. 36049219 PMC10235192

[25] Tiisanoja A., Syrjälä A.M., Tertsonen M., et al. Oral diseases and inflammatory burden and Alzheimer’s disease among subjects aged 75 years or older. Spec. Care Dentist. 2019; 39: 158–165. doi: 10.1111/scd.12357. 30693967

[26] Guo H, Chang S, Pi X, et al. The Effect of Periodontitis on Dementia and Cognitive Impairment: A Meta-Analysis. Int J Environ Res Public Health. 2021; 18: 6823. doi: 10.3390/ijerph18136823. 34202071 PMC8297088

[27] Shai I, Jiang R, Manson JE, et al. Ethnicity, obesity, and risk of type 2 diabetes in women: a 20- year follow-up study. Diabetes Care. 2006; 29: 1585–1590. doi: 10.2337/dref6-0057. 16801583

[28] Ciesielska N, Sokołowski R, Mazur E, Podhorecka M, Polak-Szabela A, Kędziora-Kornatowska K. Is the Montreal Cognitive Assessment (MoCA) test better suited than the Mini-Mental State Examination (MMSE) in mild cognitive impairment (MCI) detection among people aged over 60? Meta-analysis. Psychiatr Pol. 2016; 50: 1039-1052. doi: 10.12740/PP/45368. 27992895

